# Impact of Selected Starters and Cassava Varieties on the Proximate, Rheological, and Volatile Profiles of Lafun

**DOI:** 10.3390/foods14040660

**Published:** 2025-02-15

**Authors:** Abosede O. Fawole, Kimon-Andreas G. Karatzas, Jane K. Parker, Colette C. Fagan

**Affiliations:** 1 Department of Food and Nutritional Sciences, University of Reading, P.O. Box 226, Whiteknights, Reading RG6 6AP, UK; fawole.abosede@polyibadan.edu.ng (A.O.F.); k.karatzas@reading.ac.uk (K.-A.G.K.); j.k.parker@reading.ac.uk (J.K.P.); 2 Biology Department, The Polytechnic, Ibadan 200284, Nigeria

**Keywords:** lactic acid bacteria, cassava, proximate, fermentation, volatile flavour profile, rheological properties

## Abstract

Spontaneous fermentation is currently used to produce lafun from cassava, leading to inconsistent product quality and decreased safety. Using starter cultures and optimising the selection of the raw materials can overcome this. This study evaluated the impact of various lactic acid bacteria (LAB) starters and varieties of cassava (bitter: IBA30527; vitamin A fortified bitter: IBA011371; and sweet: TMEB117) on the proximate, rheological, and volatile profiles of lafun. The varieties were fermented with four selected LAB (two strains of *Weissella koreensis*, *Lactococcus lactis*, and *Leuconostoc mesenteroides*). The use of fortified cassava showed higher potential to improve the quality of lafun. The combination of fortified cassava and *Leuconostoc mesenteroides* gave the highest nutritional value (ash: 4.37% cf. 1.33%; protein: 3.08% cf. 0.87%; and fibre: 7.43% cf. 1.43%). Fermenting the fortified cassava with *Weissella koreensis*-2 produced lafun gruel with the best viscoelastic properties, indicating an overall better product quality. The fortified cassava fermented with combined cultures of *W. koreensis*-1 and *L. lactis* resulted in a product with lower levels of carboxylic acids (cheesy) and lipid oxidation products (fried, rancid) but higher concentrations of carotenoid-derived compounds (fruity). The use of LAB in the controlled fermentation of fortified cassava could be a sustainable alternative to improve the physical, nutritional, and flavour properties of lafun.

## 1. Introduction

Fermentation makes a valuable contribution to the development of the flavour, texture, and overall acceptability of fermented products. The concentration and composition of volatile flavour compounds can be key to consumer acceptability and preference [1]. Fermentation by microorganisms could also result in a significant increase in the quality of protein, vitamins and other essential nutrients [2]. Unlike other preservation methods focusing mainly on inhibiting the growth of microbes in food or at least reducing their numbers, fermentation encourages the growth of desired organisms whose metabolic activities improve the sensory and physical attributes of the product, in addition to extending shelf life [3,4].

Lafun is a cassava product traditionally prepared by steeping peeled cassava root pieces in water followed by fermentation for 2–5 days. The fermented roots are sun-dried on surfaces such as cement floors, tarred roads, and rocks and subsequently milled into powder [5]. Drying can take days or weeks, depending on the weather [6]. The coarse flour obtained is sieved and made into a gruel in boiling water and consumed with a stew of vegetables and a protein source. If properly stored, the shelf life of the cassava flour can be 6 months or more. Rheological analysis can reveal some of the flour qualities by estimating the degree of viscosity and deformability. A relatively high degree of viscosity and deformability of lafun gruel indicates good quality.

Rheological evaluation of food is an important tool for the assessment of product quality. Rheological measurement investigates how food materials respond to an external (applied) force (stress) and deformation (strain) as a function of time [7]. Oscillatory rheology, in which a small strain is applied, is well suited to the evaluation of the physical properties of food products, such as hydrated lafun, that are viscoelastic [8]. How a food material deforms during handling or consumption is typically a function of both the texture and rheology of that food [9]. Rheological analysis is important to monitor the effects of compositional and processing parameters that are needed in developing lafun with the desired rheological behaviour.

Since lafun is a product of spontaneous fermentation, the microbiological composition is of a mixed culture that includes *Saccharomyces cerevisiae*, *Kluyveromyces marxianus*, *Pichia scutulata*, *Lactobacillus fermentum*, *Lactiplantibacillus plantarum*, and *Bacillus cereus* [10,11].

Spontaneous fermentation can result in variable product quality and potentially the presence of microorganisms that could be detrimental to human health. Utilising food grade starter cultures in a controlled way would overcome this challenge. Previous work has been carried out to understand the predominant microorganisms in the fermentation of cassava to produce lafun and their role in the process [12,13]. Padonou et al. [5], in their work, showed the effect of combining yeast and LAB as a mixed starter culture to produce lafun.

LAB strains vary in their metabolic characteristics, impacting their capacity to degrade components in cassava and synthesise new substances, which can impact the composition, rheological properties, and volatile flavour profile of lafun. Additionally, the composition of different cassava varieties varies. Therefore, substrate variability also results in the variability of lafun composition, impacting stability, rheological characteristics, and flavour profile.

Still, there is little or no information on the impact of LAB strains and cassava varieties on the rheological properties, proximate composition, and volatile compounds of lafun. Therefore, the present study determines the impact of selected LAB strains as monoculture or co-culture on the physical properties and aroma profile of lafun produced using three varieties of cassava: bitter (B), sweet (S), and vitamin A fortified (F), in order to assess their potential as starter cultures for lafun production.

## 2. Materials and Methods

### 2.1. Collection of Raw Cassava Roots and Lafun Market Samples

Cassava roots, bitter cassava (IBA30527), sweet cassava (TMEB117), and a vitamin A-enriched yellow fortified cassava (IBA011371) were imported from the International Institute of Tropical Agriculture (IITA), Nigeria, to produce lafun. These cassava roots were harvested and waxed (to preserve the roots and maintain their freshness until used) on the day of export and shipped by air freight to the University of Reading (UoR), arriving on the third day after harvest. A lafun sample (in flour form), produced by indigenous food manufacturers and collected from Bodija market in Ibadan, Nigeria, was used as a market sample MKT1 (control). Two more market samples (flour), MKT2 and MKT3, were purchased from Songo and Mokola markets, respectively, and used in addition to MKT1 as the controls during the analysis of volatile compounds.

### 2.2. Preparation of Cassava Roots

The cassava roots were prepared according to good manufacturing practices in the food processing pilot plant at the University of Reading. The samples were washed thoroughly, peeled, and rewashed after being cut into pieces of approximately 2 cm. The pieces were packed into Ziploc polyethylene bags and blast frozen before storage at −18 °C. For lafun production, the frozen roots were initially blanched for 2 min to reduce microbial loads and cooled under a UV light cabinet with a fan before fermentation. Samples of raw cassava roots from each variety were also freeze-dried over 48 h without fermentation (CoolSafe 4–15 L Freeze Dryer, Labogene, Allerød, Denmark), milled, packed in 250 mL sterile bottles, and stored at −18 °C until further analysis.

### 2.3. Preparation of Lactic Acid Bacteria Starter Cultures

The four LAB strains used in this study, *Weissella koreensis*-1 (K1), *Lactococcus lactis* (L), *Weissella koreensis*-2 (K2), and *Leuconostoc mesenteroides* (M), were collected as stock cultures from the Food Microbiology Unit of the Department of Food and Nutritional Sciences (University of Reading, UK). They had been previously isolated from the spontaneous fermentation of cassava. In the controlled fermentation of cassava, these strains reduced pH from 7.68–7.65 to 5.02 ± 0.51 (*Weissella koreensis*-1), 4.43 ± 0.03 (*Lactococcus lactis*), 4.68 ± 0.21 (*Weissella koreensis*-2), and 4.72 ± 0.21 (*Leuconostoc mesenteroides*) in 48 h. Each strain was preserved in 2 mL cryo vials of 1860 µL cultured broth and 140 µL of dimethyl sulfoxide at −80 °C. They were reactivated by plating on either De Man, Rogosa, and Sharpe (MRS) agar (strains K1, K2, and M) or M17 agar (strain L). Incubation was performed anaerobically at 37 ± 1 °C for 48 h.

### 2.4. Lafun Production

The selected LAB starter cultures were used in the controlled fermentation of the prepared cassava roots singly and as a co-starter culture (four combinations were chosen based on the rate of acidification in a previous study). The procedure used for the three cassava varieties was similar. Samples of prepared roots (300 g) were steeped in 300 mL of sterile water. Samples were inoculated singly with 3000 µL of cultured broth of each strain (8.0 log CFU ml^−1^). In the co-starter culture, 1500 µL of each of the cultured broths of the two LAB strains was combined and used. These were placed in an anaerobic workstation (MG1000, Don Whitley Scientific, Bingley, UK) at 37 ± 1 °C for 48 h to ferment. The samples were taken out after 48 h, frozen to −20 °C, and freeze-dried for 4 days in a CoolSafe 4–15 L Freeze Dryer at −55 °C (LaboGene, UK). Dried samples were milled with a coffee blender. The flour obtained (*n* = 24) was packed into a 250 mL sterile bottle and stored at −18 °C for further analyses.

### 2.5. Proximate Analysis of Lafun Samples and Raw Cassava Roots

The laboratory samples (four strains × three cassava types; two batches gave *n* = 24), market sample (MKT1), and raw cassava roots were analysed for moisture, ash, crude protein, crude lipid, crude fibre, and digestible carbohydrates. The proximate analysis was carried out in duplicate and on a dry weight basis using AOAC [14] methods. The moisture content was determined via a thermogravimetric method. A sample (3 g) was dried in an oven at 100 °C to a constant weight. The ash content was determined gravimetrically by pre-ashing a sample (3 g), which was then transferred to a muffle furnace at 550 °C for 8 h. The crude protein was determined using the Kjeldahl method. The crude lipid content was determined via Soxhlet extraction. The crude fibre content was determined via acid detergent fibre analysis. The estimation of digestible carbohydrates present in lafun was calculated by the difference after the analysis of all the other items in the proximate analysis.

### 2.6. Lafun Gruel Rheological Measurements

Lafun gruel was prepared from laboratory samples (*n* = 24) and the market sample (MKT1) by stirring 7 g of the samples into 30 mL of water (95 °C) until a gruel was formed. The gruel was prepared immediately prior to analysis. The analysis was performed using a dynamic oscillatory test through the use of a controlled stress-strain rheometer (Anton Paar MCR 102, Ostfildern, Germany) and a 50 mm serrated parallel plate. The lafun gruel samples were then placed between the serrated plates, and the gap was set at 1 mm; the edges were trimmed with a spatula. The samples were rested between the plates for 1 min before testing. The measurements were performed at a constant temperature of 25 °C using a Peltier plate. For all measurements performed within a specific viscoelastic region, amplitude sweeps were completed between the range of 0.0001 and 100%. Oscillation stresses for lafun gruels were then selected from the result of the amplitude sweeps test. Frequency sweep tests were set up at frequencies between 0.1 and 10 Hz with a percentage strain of 0.1 in the viscoelastic region. The storage modulus (G’) and loss modulus (G”) were then calculated using the manufacturer’s software. Each sample was analysed in triplicate with the results presented as a mean.

### 2.7. Volatile Compound Analysis of Lafun Samples and Raw Cassava Roots

#### 2.7.1. Sample Preparation

For gas chromatography–mass spectrometry (GC-MS) analysis, laboratory samples (8 strains × 3 cassava × 2 batches) and 3 market samples and raw cassava roots from each of the 3 varieties (total *n* = 54) were prepared by adding 3 mL of standard solution (200 μg L^−1^ thymol in saturated sodium chloride; NaCl) to 0.5 g of lafun in 20 mL glass SPME vials. Samples were then mixed thoroughly using a vortex mixer (MS1, Minishaker, UK) for 30 s. The analysis was run in triplicate. For gas chromatography–olfactometry (GC-O), one of the lafun samples collected from the markets and one processed in the laboratory (0.5 g) were used. This sample was selected based on the high number of volatiles and their concentrations. Each sample was prepared by adding 3 mL of saturated NaCl and mixed thoroughly using a vortex.

#### 2.7.2. Gas Chromatography–Mass Spectrometry Analysis

The volatile compounds were extracted by solid-phase microextraction (SPME) using a 50/30 μm DVB/CAR/PDMS Stableflex fibre (Supelco, Poole, UK). The prepared samples were equilibrated for 10 min at 35 °C with agitation (500 rpm). The SPME fibre was then exposed to the headspace for 30 min, followed by desorption in the GC injection port (splitless) at 250 °C. An Agilent 5975C Series GC/MSD coupled to an Agilent 7890A gas chromatograph (Agilent, Santa Clara, CA, USA) was used, equipped with a Zebron DBWax column (30 m × 250 μm × 0.5 μm, Phenomenex, Macclesfield, UK). The oven was held at 40 °C for 5 min, and the temperature was subsequently increased to 250 °C at a rate of 4 °C min^−1^ and held at 250 °C for 5 min. The carrier gas was helium at a flow rate of 0.9 mL min^−1^. Mass spectra were recorded in electron impact mode at an ionisation voltage of 70 eV and a source temperature of 230 °C. A scan range of *m*/*z* 20–280 with a scan time of 0.69 s was employed, and the data were collected and stored using the ChemStation system. Volatiles were identified through the comparison of spectra and linear retention indices (based on C5–C26 alkane series) with those obtained from authentic compounds. Semi-quantitation was carried out based on the quantification of a key ion multiplied by a factor to account for the contribution from all ions (the total ion chromatogram). These peak areas were compared with those of the internal standard using a response factor of 1.

#### 2.7.3. Gas Chromatography–Olfactometry Analysis

The prepared sample was equilibrated with agitation at 35 °C for 10 min. The SPME fibre was then exposed to the headspace, just above the sample, for 30 min by penetrating the sample bottle liner with the stainless-steel needle housing the fibre. The extract was analysed using a DB-5 column (30 m length × 0.25 mm diameter × 0.25 μm thickness). Analysis was performed by releasing the fibre into the injection port of an Agilent 7890B gas chromatograph (Agilent Technologies, Santa Clara, CA, USA) fitted with an ODO II GC–O system (SGE Ltd., Ringwood, Australia). The outlet was split (ratio of 1:1) between a sniffing port and a flame ionisation detector. The temperature of the injector and detector was maintained at 250 °C. The oven temperature started at 40 °C and increased at 4 °C min^−1^ to 250 °C. Helium (carrier gas) had a flow rate of 1.2 mL min^−1^. n-Alkanes C5–C25 were analysed to find linear retention index (LRI) values for the odour active components. Detection and verbal description of the odour active components were carried out in duplicate by two experienced assessors, scoring on a scale where 3 = weak, 5 = medium, and 7 = strong.

### 2.8. Statistical Analysis

Data from proximate and rheological analyses were expressed as mean ± standard deviation. One-way analysis of variance (ANOVA) and post hoc Tukey’s HSD were used to determine statistical difference (*p* < 0.05). Principal component analysis (PCA) was used to analyse the volatile data. The XLSTAT 2018.1 software package was used for all the statistical analyses.

## 3. Results and Discussion

### 3.1. Proximate Analysis of Lafun Samples and Raw Cassava Roots

Fermented cassava products are important sources of carbohydrates; this was reflected in the percentage of carbohydrates estimated in all the samples (Table 1). Cassava root generally has a higher carbohydrate concentration compared to foods such as potatoes [15]. According to the literature, about 72% of this is starch in the form of amylopectin and amylose. The presence of sucrose, fructose, and glucose has also been reported [16]. The main sugar in cassava is glucose, with low levels of galactose, xylose, rhamnose, arabinose, and mannose according to [17]. The proximate composition of lafun samples from the fermentation process revealed a higher level of crude fibre (the highest being 7.4%) compared to the market sample (1.4%). The higher values of fibre content recorded in the laboratory lafun samples compared to the market samples could be due to the controlled fermentation employed in the former.

Crude fibre can have positive effects on health as it influences the digesta pH and transit rate through the digestive tract, improving intestinal health [18]. Food transit is crucial for digestion and absorption of nutrients, appetite regulation, gut hormone signalling, and gut microbiota metabolism [19]. Thus, a good level of fibre in diets improves glucose tolerance, prevents constipation, and reduces cholesterol levels [20].

There was no significant difference (*p* > 0.05) in the moisture content between most of the samples (Table 1). The similar values in the moisture content of the laboratory samples indicate that the drying stage had been well controlled. However, there were significant differences (*p* < 0.05) in ash, protein, lipid, and fibre across the tested samples. The ash content of the raw bitter cassava (1.8%) was significantly higher than that of all the lafun samples produced from this variety (0.8% to 1.7%). The same trend was seen in the sweet variety (2.3%) and its products, except the samples produced with *L. mesenteroides* (2.4%) and the co-cultures of the two *W. koreensis* strains (3.0%) and *W. koreensis*-1 with *L. lactis* (3.2%). However, the values of ash in the lafun produced from fortified cassava (3.4% to 4.4%) were significantly higher than those of the raw material (3.0%).

Eromosele et al. [21] reported that the increase in the ash level after fermentation could be the result of an incomplete utilisation of the nutrients present in the raw material by the fermenting organisms. The fortified cassava is a vitamin A-enriched ‘yellow’ cassava that provides more vitamin A in the diets. Thus, the increase in the ash content recorded after fermentation of the fortified cassava could be the result of an incomplete utilisation of vitamin A.

The lipid content of the raw bitter cassava is similar to that of its lafun products (0.5%) and statistically different from the rest of the cassava varieties (0.4% to 0.6%). The lipid contents of raw fortified cassava and sweet cassava (1.4% and 0.7%, respectively) were significantly different from those of their lafun products, ranging from 0.8% to 2.2% and 0.2% to 1.1%, respectively. In cases where there was a reduction in lipid content after fermentation, this could be advantageous in decreasing the chance of rancidity in hydrated lafun [22]. Chikwendu et al. [23] fermented pearl millet, like the former author, but recorded an increase in lipid content after fermentation. The opposing observation (increase or decrease in lipid content after fermentation) in both studies could suggest that the proximate composition of products of a fermented plant is influenced by the type of variety of that plant used. Samples with an increased lipid level might require additional storage controls to extend their shelf life.

There was a significant increase (*p* < 0.05) in the protein content of lafun (2.5% to 3.1%) from fortified cassava relative to the raw material (1.8%). Previous studies have proven that fermentation can increase protein content in food [21,24,25]. However, Oyewole and Odunfa [26] reported results opposing the above (reduction in protein level after fermentation), following experiments with both bitter and sweet cassava lafun products. The reduction in protein content after the fermentation of bitter and sweet cassava varieties used could be an indication that the varieties have more sulphur amino acid content that might have been metabolised by the LAB starters [27]. Since lafun is never consumed alone, its lack of protein is compensated for by consuming it with soup and meat that are high in protein.

It is interesting to note that the highest values in the ash (4.4%), protein (3.1%), and fibre (7.4%) contents were in fortified samples fermented with *L. mesenteroides*. There is limited data available regarding the role of *L. mesenteroides* as a starter in the improvement of the proximate values of fermented cassava products or food in general. Tefera et al. [28] reported the ability of *L. mesenteroides* to reduce cyanide in fermented cassava flour. There are also some reports on its excellent role in probiotic and antimicrobial activities and its impact on the texture and flavour quality of some other fermented foods, such as curd, kimchi, and fermented cabbage [29,30,31].

### 3.2. Lafun Gruel Rheological Properties

**Figure 1:**
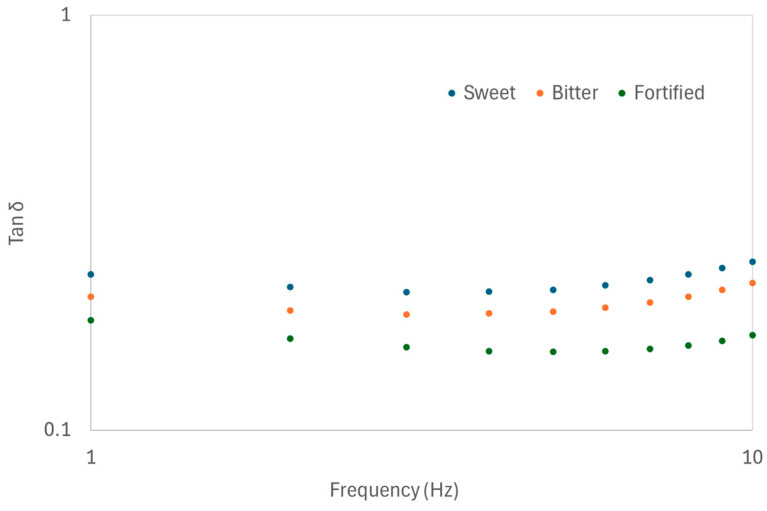
Tan *δ* as a function of the frequency of lafun gruel produced using *L. mesenteroides*, typical of fortified (green), bitter (orange), and sweet (blue) cassava varieties (bitter: IBA30527; fortified: IBA011371; and sweet: TMEB117).

Frequency sweep studies (a dynamic test) were conducted to characterise the rheological properties of the experimentally produced lafun samples (Table 2). The storage modulus G’ and the loss modulus G” were obtained from a rheological test in the linear viscoelastic range. G’ is an amount of the deformation energy stored in the sample during the shear process (elastic behaviour of the sample), and G” indicates the deformation energy sapped during shear but lost to the sample afterward (viscous behaviour of the sample) [8]. The plots of frequency against both G’ and G” produced a linear relationship in all the samples (G’, R^2^ ≥ 0.80 and G”, R^2^ ≥ 0.90). Regardless of the treatment used, G’ was always higher than G”, signifying that lafun gruel exhibited more solid-like behaviour [8]. Also, the values of the two moduli increased with increasing frequency from 0.1 Hz to 10 Hz. To further understand the inherent attribute of the bonds within each sample, tan *δ* (G”/G’) was plotted against frequency. Figure 1 shows the impact of cassava variety on the tan *δ* of the lafun sample prepared using *L. mesenteroides* as the starter culture, and a similar trend was observed for all other samples. Tan *δ* values for all the samples were hardly affected by frequency. However, tan *δ* values for both bitter and sweet cassava were higher, which indicates that they are more viscous than the fortified variety and have a greater number of weak-bond interactions within the gruel [32].

The more elastic the samples are, the higher the consumer acceptability, as lafun is a food product expected to behave like an elastic material based on the characteristics mentioned by [33]. Thus, the behaviour of the laboratory lafun samples was compared with that of the market sample. The G’ values obtained for the market sample were higher only in the lafun samples produced with bitter cassava and *W. koreensis*-2 and a combination of both *W. koreensis* strains starters, as well as the sample produced with sweet cassava and *L. mesenteroides*. It can, therefore, be inferred that 88% of the laboratory samples had higher deformability and thus exhibited elastic-like behaviour (Table 2). These results suggest that the majority of the laboratory samples have better quality regarding rheological properties than the market samples studied. This suggestion is based on the standard rheological quality of hydrated flour, which is, high deformability. Laboratory lafun flour showed better lafun gruel-making behaviour and resulted in superior lafun gruel quality.

Additionally, rheological measurement revealed different trends in the behaviour of gruel lafun made with different cassava varieties. Thus, the rheology of lafun was primarily affected by the type of cassava used. This behaviour agrees with the findings of Hüttner et al. [34], who reported that different flour characteristics influence rheological properties. The products of fortified cassava are more elastic than the products of the other two varieties. This solid-like behaviour of fortified cassava products could be an indication of a better water-holding capacity and, therefore, better product quality.

### 3.3. Volatile Compound Analysis of Lafun Samples and Raw Cassava Roots

#### 3.3.1. Gas Chromatography-Mass Spectrometry Analysis

In this study, SPME-GC–MS was used for the characterisation of key volatile aroma compounds in lafun. The identification and quantitation of 35 volatile organic compounds (VOCs) from lafun samples (*n* = 51) and raw cassava roots (*n* = 3) on a dry weight basis was possible using Solid Phase Microextraction (SPME). The VOCs selected for semi-quantitation include a total of 16 aldehydes, 2 carotenoid derivatives, 5 acids, 1 alcohol, 1 furan, 9 ketones, and 2 phenols (only found in the Sango market sample).

**Figure 2:**
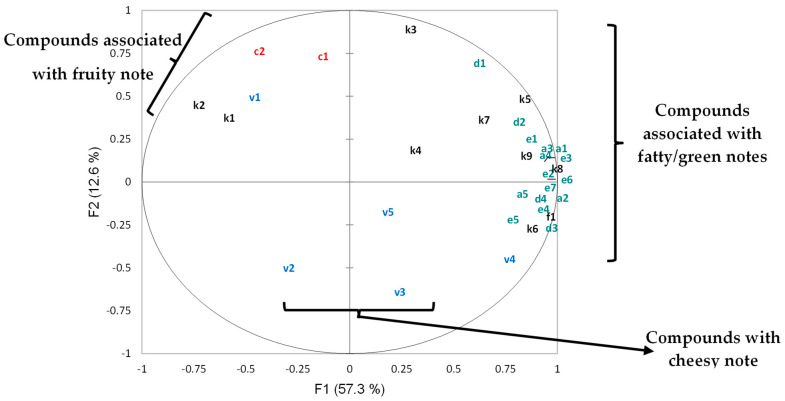
Principal component plot of volatile compounds (variables: aldehydes, ketones, organic acids, furan), PC1 vs. PC2: red is carotenoid-derived; blue is acid; green is lipid degradation product, and black is ketone. The two components account for 69.9% of the total variance. See Table 3, Table 4 and Table 5 for volatiles codes.

PCA analysis was performed to determine the impact of the starter cultures and cassava varieties on the identified volatile compounds. PC1 and PC2 accounted for 69.9% of the total variance in the VOCs (Figure 2). The principal components separated samples into three groups: one containing bitter and sweet cassava varieties, the second containing the fortified cassava variety, and the final group containing the market samples at the bottom left side. The variables that determine this separation are aldehydes, ketones, and volatile acids. The lafun samples produced with both bitter and sweet cassava varieties had more abundant lipid degradation compounds (a1–a5; d1–d4 and e1–e7). Those from fortified cassava were richer in carotenoid derivatives (c1 and c2), consistent with their high vitamin A content. The market samples were mainly driven by volatile acids (v2, v3, and v5). The fortified cassava samples had fewer lipid degradation compounds for all cultures, suggesting that it is less prone to the development of rancidity.

**Figure 3:**
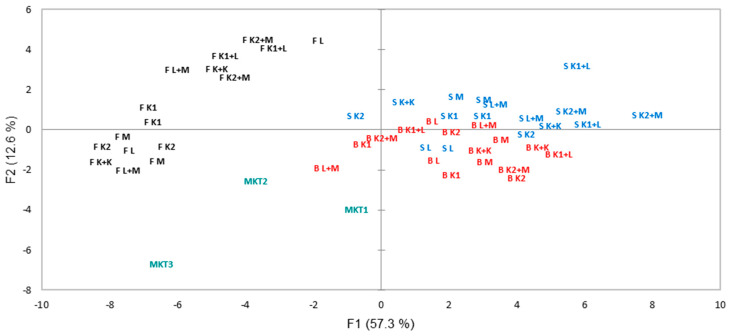
Projection of lafun produced with bitter cassava-IBA30527 (red), sweet cassava-TMEB117 (blue), and fortified-cassava IBA011371 (black) with market samples (green) onto the two principal components. B = bitter, S = sweet, F = fortified, L = *L. lactis*, K1 = *W. koreensis*-1, K2 = *W. koreensis*-2, M = *L. mesenteroides*.

The PCA score plot of volatile compounds showed that the samples from fortified cassava were considerably different from the others along PC1, explaining 57.3% of the variance (Figure 2). The PCA plot of volatile compounds also showed that 6-methyl-5-hepten-2-one, 6,10-dimethyl-5,9-undecadien-2-one, acetic acid, acetone, and 2-butanone were closely correlated with lafun from fortified cassava (Figure 3). The correlation of 6-methyl-5-hepten-2-one and 6,10-dimethyl-5,9-undecadien-2-one with the fortified cassava could be as a result of the carotenoids present in this variety. This is reasonable, as these compounds are known to be degradation products or oxidative by-products derived from carotenoids. Carotenoid pigmentation has been reported to affect the volatile composition of some plants [35]. On the other hand, butanoic acid correlated with all market lafun samples, while the majority of identified lipid-degrading compounds were closely correlated to lafun from both sweet and bitter cassava. Organic acids such as heptanoic, hexanoic, and pentanoic acids were related to the negative axis of the PC2 and were correlated to most of the bitter cassava products.

The volatile profile of lafun fermented with LAB cultures varied greatly among strains and with cassava varieties. Lipid-derived aldehydes are prevalent components of food products. The aldehyde compounds identified in lafun could originate either from the enzymatic or chemical oxidation of lipids [36]. As pointed out by Kazeniac and Hall [37], the enzymatic oxidation of lipids yields a wide range of carbonyl compounds, among which are acetone, pentanal, hexanal, heptanal, octanal, nonanal, (E)-2-pentenal, (E)-2-hexenal, (E)-2-heptenal, (E)-2-octenal, (E)-2-nonenal, (E)-2-decenal, (E)-2-undecenal, (E,E)-2,4-heptadienal, and (E,E)-2,4-nonadienal. All of these were also found in this study (Table 3 and Table 4).

Hexanal was particularly high in the sample of bitter cassava produced with the combination of *W. koreensis*-1 and *L. lactis*. Hexanal is one of the many well-documented aromatic compounds in the literature. It contributes to flavour and aroma, and it is used as a measure of the oxidative status of foods and antimicrobial properties [38,39]. Like pentanal and 3,5-octadien-2-one, hexanal is a product of autoxidation. Hexanal has been found to be formed during the termination phase of specific fatty acids oxidation (linoleic and arachidonic acids). Therefore, it is used as a measure of oxidative stability [40]; however, it is the alkadienals that are often the most potent and give a rancid note.

The oxidation of lipids is the cause of rancidity in food and thereby its reduction in quality [41,42]. The lipid content of lafun from fortified cassava is generally higher than those of the other two varieties, as noted in the proximate analysis, but their hexanal, pentanal, and 3,5-octadien-2-one are much lower. Therefore, lipid oxidation is not related to lipid content, but to the lipid profile of the sample, the oxidative status of the sample, or the antioxidant activity of vitamin A. In this study, the most likely cause is related to the antioxidant activity of vitamin A. Among the organic acids identified, acetic acid had the highest concentration in lafun. This could be related to the high content of acetic acid produced during fermentation [43].

Phenols were only found in the Sango market sample (Table 5). p-Cresol can be formed by the degradation of tyrosine and has a characteristic medicinal or coal-tar smell. However, it is also released into the environment through fuel combustion (wood and trash burning and vehicle exhausts). A small number of cresols have been detected in foods such as tomatoes, asparagus, butter, and some drinks such as coffee, brandy, black tea, and wine [44]. The presence of cresols in the market sample could be due to the process used during the drying stage. Some indigenous producers dry lafun by spreading it on tarred roads that could contain cresol and where automobile exhaust emissions are heavy. Cresols are toxic but unlikely to cause harm at such low levels.

#### 3.3.2. Gas Chromatography–Olfactometry Analysis

A total of 50 odorants were detected by both assessors by GC-O analysis of the samples (Table 6), 27 of which were detected in the lafun samples, with the remaining 23 being highly potent aroma compounds present at levels below the instrumental detection limit. All but 5 of these were identified; 37 were confirmed with authentic standards, and 8 were identified using the LSB@TUM odorant database or other literature sources. Lipid-derived aldehydes had the highest odour intensities, contributing mainly green, fried, and coriander odours. However, 3-hexenal and 6-methyl-5-hepten-2-one contributed to the desirable sweet and orange notes in both samples. It was clearly observed that (E,E)-2,4-decadienal (fried note) had the highest intensity in both samples. Quantitative differences were observed between the market sample and the laboratory sample. The most notable difference was the contribution of an off-note from methoxypyrazines, p-cresol, and guaiacol, found only in the market sample. Furthermore, butanoic acid was not detected in the GC-O analysis of the lafun produced by LABs. There are no data in the literature regarding odour description in lafun, and the identified odourants were detected for the first time in this study.

To summarise, aldehydes occurred mostly among the odour-active compounds found in lafun, as mentioned earlier. The shorter-chain aldehydes (<C7) are important contributors to the characteristic ‘fresh green’ odour of vegetables. They are widely used as food additives because of this fresh green odour [45]. The longer chain and more unsaturated aldehydes are very potent. They are responsible for the rancid notes in many foods and reduce their keeping quality. The results of this study indicate the need for an effective storage method that will lower the rate of oxidation of aldehydes in lafun. The oxidation of aldehydes may lead to the corresponding organic acids [46]. The high concentration of organic acids in the market samples could result from the oxidation of aldehydes. Butanoic acid, characterised by an unpleasant odour of rancid cheese, was very high in both the Sango (6097 µg kg^−1^) and Mokola (5653 µg kg^−1^) market samples. The GC-MS results of butanoic acid showed that the production method of the laboratory samples is a good tool for reducing unwanted volatile organic compounds, such as butanoic acid, to a very low level. In addition, the absence of cresols in the laboratory samples could be related to the fact that they were not dried on the road like the market samples. The absence of off-note odour due to the absence of methoxypyrazine in laboratory samples is another indication of high-quality processing achieved in lafun.

## 4. Conclusions

The lactic acid fermentation of cassava has excellent potential to produce high-quality lafun. The present study demonstrates that the quality of lafun produced will vary depending on the starter culture and cassava variety used. In particular, the characteristics of the cassava variety employed in lafun production clearly affect the textural and organoleptic properties of the lafun, as well as the overall eating quality. For the first time, it has been demonstrated that the inoculation of fortified cassava (IBA011371) with *L. mesenteroides* starter culture strain results in higher crude ash, protein, and fibre content in the lafun, and this provides a product with more elasticity, which indicates improved eating quality. Bitter cassava is traditionally used to produce lafun; however, the results of this study provide evidence that using fortified cassava roots to manufacture lafun results in more desirable rheological properties, suggesting that the use of fortified cassava could improve consumer acceptability. Carotenoid-derived compounds in the headspace of lafun were found by GC-MS to be generally higher in the fortified variety products than the other two varieties, while the compounds typically responsible for rancid and cheesy notes were lower in this variety. The fermentation process should be controlled by parameters that would minimise lipid oxidation and increase the yield of the carotenoid compounds for a better lafun flavour and quality. The hexanal level in food during storage should be monitored to determine the onset of rancidity. A storage study should be conducted to check the effect of oxidation on the changes of the lafun flavour profile.

## Figures and Tables

**Table 1:** Percentage proximate composition of lafun flour produced with LAB starter cultures, three cassava varieties (raw) (bitter: IBA30527; fortified: IBA011371; and sweet: TMEB117) and Bodija market sample.

		% Proximate					
LAB Strain	Cassava Variety	Moisture Content	Ash	Crude Protein	Crude Lipid	Crude Fibre	Carbohydrate
Unfermented (Raw Cassava)	Bitter	2.3 ± 0.0 ^bc^	1.8 ± 0.2 ^fghij^	1.8 ± 0.1 ^de^	0.5 ± 0.14 ^fgh^	2.1 ± 0.04 ^hi^	91.5 ^cdefg^
	Fortified	2.3 ± 0.5 ^bc^	3.0 ± 0.0 ^de^	1.8 ± 0.1 ^de^	1.4 ± 0.01 ^bcd^	3.4 ± 0.03 ^efg^	88.1 ^hi^
	Sweet	2.2 ± 0.2 ^bcd^	2.3 ± 0.0 ^efg^	2.2 ± 0.0 ^cd^	0.7 ± 0.14 ^efg^	1.4 ± 0.01 ^k^	91.2 ^defg^
K1	Bitter	1.4 ± 0.1 ^bcd^	1.3 ± 0.1 ^hij^	1.1 ± 0.0 ^defg^	0.4 ± 0.00 ^gh^	3.1 ± 0.05 ^g^	92.7 ^abc^
	Fortified	2.3 ± 0.5 ^bc^	3.8 ± 0.2 ^abc^	3.0 ± 0.2 ^ab^	2.2 ± 0.00 ^a^	3.5 ± 0.10 ^efg^	85.2 ^hi^
	Sweet	0.9 ± 0.1 ^cd^	1.5 ± 0.2 ^ghi^	1.4 ± 0.0 ^cdef^	0.2 ± 0.00 ^h^	2.5 ± 0.26 ^h^	93.5 ^a^
L	Bitter	1.2 ± 0.2 ^bcd^	1.5 ± 0.2 ^hij^	0.9 ± 0.0 ^fg^	0.5 ± 0.14 ^fgh^	3.6 ± 0.25 ^defg^	92.4 ^abcd^
	Fortified	1.9 ± 0.7 ^bcd^	3.8 ± 0.3 ^abc^	2.9 ± 0.1 ^ab^	1.7 ± 0.14 ^b^	6.6 ± 0.11 ^b^	83.1 ^ij^
	Sweet	1.0 ± 0.90 ^bcd^	1.5 ± 0.19 ^ghi^	1.4 ± 0.07 ^cdef^	0.4 ± 0.00 ^gh^	2.1 ± 0.07 ^hi^	93.5 ^a^
K2	Bitter	0.9 ± 0.38 ^bcd^	1.3 ± 0.09 ^hij^	1.5 ± 0.05 ^cde^	0.4 ± 0.00 ^gh^	1.9 ± 0.00 ^ij^	94.0 ^a^
	Fortified	2.3 ± 0.47 ^bc^	3.6 ± 0.05 ^bcd^	2.9 ± 0.06 ^ab^	1.5 ± 0.14 ^b^	4.8 ± 0.08 ^c^	84.9 ^hi^
	Sweet	1.5 ± 0.71 ^bcd^	1.4 ± 0.05 ^hij^	1.3 ± 0.17 ^cdef^	1.1 ± 0.13 ^cd^	3.7 ± 0.22 ^def^	91.0 ^bcde^
M	Bitter	1.5 ± 0.19 ^bcd^	1.5 ± 0.19 ^hij^	0.9 ± 0.04 ^fg^	0.6 ± 0.00 ^fg^	3.2 ± 0.21 ^fg^	92.3 ^abcd^
	Fortified	1.8 ± 0.28 ^bcd^	4.4 ± 0.05 ^a^	3.1 ± 0.23 ^a^	1.4 ± 0.00 ^bc^	7.4 ± 0.18 ^a^	81.9 ^j^
	Sweet	0.7 ± 0.09 ^d^	2.4 ± 0.33 ^ef^	1.5 ± 0.05 ^cde^	0.6 ± 0.00 ^fg^	3.4 ± 0.05 ^efg^	91.3 ^bcde^
L + M	Bitter	1.2 ± 0.61 ^bcd^	1.7 ± 0.10 ^fgh^	1.1 ± 0.01 ^efg^	0.5 ± 0.14 ^fgh^	3.4 ± 0.01 ^efg^	92.1 ^abcd^
	Fortified	1.7 ± 0.42 ^bcd^	3.9 ± 0.12 ^ab^	2.9 ± 0.05 ^ab^	1.7 ± 0.14 ^b^	6.7 ± 0.13 ^b^	83.1 ^ij^
	Sweet	1.1 ± 0.14 ^bcd^	2.2 ± 0.28 ^fg^	1.6 ± 0.10 ^cde^	0.5 ± 0.14 ^fgh^	4.1 ± 0.09 ^d^	90.5 ^de^
K + K	Bitter	2.2 ± 0.24 ^bcd^	0.8 ± 0.24 ^j^	0.8 ± 0.06 ^g^	0.4 ± 0.00 ^gh^	2.3 ± 0.07 ^hi^	93.51 ^a^
	Fortified	2.2 ± 0.28 ^bcd^	3.6 ± 0.05 ^bcd^	2.6 ± 0.09 ^ab^	1.0 ± 0.00 ^de^	3.9 ± 0.25 ^de^	86.7 ^gh^
	Sweet	2.0 ± 0.52 ^bcd^	3.0 ± 0.00 ^de^	1.8 ± 0.15 ^c^	0.4 ± 0.00 ^gh^	2.2 ± 0.01 ^hi^	90.6 ^cde^
K1 + L	Bitter	1.7 ± 0.00 ^bcd^	1.0 ± 0.00 ^ij^	0.6 ± 0.25 ^g^	0.5 ± 0.14 ^fgh^	2.1 ± 0.05 ^hi^	94.2 ^a^
	Fortified	1.8 ± 0.27 ^bcd^	3.9 ± 0.11 ^ab^	2.5 ± 0.33 ^b^	0.8 ± 0.00 ^def^	3.3 ± 0.07 ^efg^	87.6 ^fg^
	Sweet	1.3 ± 0.09 ^bcd^	3.2 ± 0.23 ^cd^	1.8 ± 0.01 ^c^	0.7 ± 0.14 ^efg^	3.7 ± 0.13 ^defg^	89.4 ^ef^
K2 + M	Bitter	2.5 ± 0.24 ^bcd^	0.8 ± 0.24 ^j^	0.8 ± 0.13 ^g^	0.5 ± 0.14 ^fgh^	2.3 ± 0.06 ^hi^	93.2 ^ab^
	Fortified	2.4 ± 0.05 ^bc^	3.5 ± 0.19 ^bcd^	2.8 ± 0.10 ^ab^	0.8 ± 0.00 ^def^	3.7 ± 0.04 ^def^	86.9 ^gh^
	Sweet	1.2 ± 0.24 ^bcd^	1.7 ± 0.09 ^gh^	1.6 ± 0.12 ^cd^	0.6 ± 0.00 ^fg^	3.6 ± 0.19 ^defg^	91.2 ^bcde^
MKT1	Market sample	10.5 ± 0.24 ^a^	1.3 ± 0.00 ^hij^	0.9 ± 0.22 ^fg^	0.5 ± 0.14 ^fgh^	1.4 ± 0.04 ^j^	85.4 ^h^

^abcdefghij^ within each column; samples with the same letter are not significantly different (Tukey’s test, *p* = 0.05); values are mean ± SD. Bitter (IBA30527), fortified (IBA011371), and sweet (TMEB117). K1 is *W. koreensis-1*; L is *L. lactis*; k2 is *W. koreensis-2*, M is *L. mesenteroides*; L + M = *L. lactis* and *L. mesenteroides*; K + K is *W. koreensis-1* and *W. koreensis-2*; K1 + L is *W. koreensis-1* and *L. lactis*; K2 + M is *W. koreensis-2* and *L. mesenteroides*; MKT1 is hydrated lafun from Bodija market as the control.

**Table 2:** Rheological properties of lafun gruel produced from hydrated lafun of control fermentation of LAB, three cassava varieties (raw) (bitter: IBA30527; fortified: IBA011371; and sweet: TMEB117), and the Bodija market sample.

LAB Strain(s)	Cassava Variety	Storage Modulus	Loss Modulus	δ Value
K1	Bitter	1378.73 ^fgh^ ± 21.19	287.89 ^cdef^ ± 4.29	0.21
	Fortified	2864.80 ^l^ ± 105.32	480.90 ^j^ ± 11.34	0.17
	Sweet	1467.57 ^gh^ ± 170.91	356.27 ^fgh^ ± 41.47	0.24
L	Bitter	1215.97 ^def^ ± 84.35	284.56 ^cdef^ ± 12.29	0.23
	Fortified	2775.27 ^kl^ ± 234.98	489.91 ^j^ ± 39.32	0.18
	Sweet	1283.63 ^efg^ ± 77.14	300.27 ^def^ ± 17.78	0.23
K2	Bitter	869.46 ^a^ ± 61.49	205.25 ^a^ ± 10.73	0.24
	Fortified	4589.87 ^n^ ± 466.45	804.57 ^l^ ± 66.35	0.18
	Sweet	999.64 ^abcd^ ± 62.95	241.09 ^abcd^ ± 9.92	0.24
M	Bitter	1260.83 ^efg^ ± 58.77	274.69 ^abcde^ ± 12.09	0.22
	Fortified	2324.40 ^i^ ± 334.78	382.09 ^ghi^ ± 58.3	0.16
	Sweet	926.81 ^abc^ ± 86.34	228.18 ^abcd^ ± 15.34	0.25
L + M	Bitter	1197.10 ^cdef^ ± 19.77	268.96 ^abcde^ ± 1.28	0.22
	Fortified	2340.33 ^ij^ ± 82.14	390.51 ^ghi^ ± 9.79	0.17
	Sweet	1329.67 ^efgh^ ± 29.93	288.32 ^cdef^ ± 3.79	0.22
K + K	Bitter	908.65 ^ab^ ± 41.81	226.29 ^abc^ ± 9.49	0.25
	Fortified	2561.83 ^jk^ ± 76.51	439.02 ^ij^ ± 13.45	0.17
	Sweet	1275.67 ^efg^ ± 61.29	282.60 ^cde^ ± 9.74	0.22
K1 + L	Bitter	1093.03 ^abcde^ ± 22.05	239.95 ^abcd^ ± 3.24	0.22
	Fortified	3480.77 ^m^ ± 159.46	621.58 ^k^ ± 16.47	0.18
	Sweet	1543.57 ^h^ ± 61.08	322.29 ^efg^ ± 10.36	0.21
K2 + M	Bitter	1303.20 ^efg^ ± 28.9	281.44 ^bcde^ ± 8.3	0.22
	Fortified	2292.30 ^i^ ± 57.57	397.48 ^hi^ ± 7.69	0.17
	Sweet	1470.27 ^gh^ ± 29.28	335.22 ^efgh^ ± 2.87	0.23
MKT1	Market sample	1130.13 ^bcde^ ± 8.2	208.07 ^ab^ ± 0.99	0.18

^abcdefghijklmn^ within each column; samples with the same letter are not significantly different (Tukey’s Test, *p* = 0.05); values are mean ± SD of three replicates. Bitter (IBA30527), fortified (IBA011371), and sweet (TMEB117). K1 is *W. koreensis-1*; L is *L. lactis*; k2 is *W. koreensis-2*, M is *L. mesenteroides*; L + M = *L. lactis* and *L. mesenteroides*; K + K is *W. koreensis-1* and *W. koreensis-2*; K1 + L is *W. koreensis-1* and *L. lactis*; K2 + M is *W. koreensis-2* and *L. mesenteroides*; MKT1 is hydrated lafun from the Bodija market as the control.

**Table 3:** Approximate relative concentration of volatile compounds µg kg^−1^ (mean ± SD, *n* = 3) quantified in lafun produced with three cassava varieties (bitter: IBA30527; fortified: IBA011371; and sweet: TMEB117) and fermented with LAB monocultures.

		*W. koreensis-1*			*L. lactis*			*W. koreensis-2*			*L. mesenteroides*		
Code	Volatile Compound	B	F	S	B	F	S	B	F	S	B	F	S
		Aldehydes											
a1	Pentanal	416 ± 93 ^abcde^	95 ± 48 ^def^	441 ± 30 ^abc^	420 ± 7 ^abcd^	269 ± 297 ^abcdef^	427 ± 88 ^abcd^	503 ± 110 ^a^	72 ± 8 ^f^	446 ± 60 ^abc^	436± 32 ^abc^	82 ± 23 ^ef^	443 ± 17 ^abc^
a2	Hexanal	12520 ± 2890 ^a^	1078 ± 16 ^b^	11117 ± 11 ^a^	11816 ± 1207 ^a^	3581 ± 4286 ^b^	11641 ± 1006 ^a^	13352 ± 1314 ^a^	315 ± 153 ^b^	11375 ± 2453 ^a^	12982 ± 323 ^a^	245 ± 96 ^b^	12154 ± 2864 ^a^
e1	2-Pentenal (E)-	139 ± 3 ^bcd^	49 ± 24 ^bcd^	240 ± 133 ^abc^	188 ± 40 ^abcd^	82 ± 85 ^bcd^	156± 23 ^abcd^	180 ± 6 ^abcd^	13 ± 18 ^bcd^	176± 40 ^abcd^	181 ± 40 ^abcd^	25 ± 1 ^bcd^	216± 21 ^abcd^
a3	Heptanal	398 ± 0 ^abc^	10 ± 13 ^c^	510 ± 102 ^abc^	349 ± 62 ^abc^	248 ± 350 ^abc^	602 ± 120 ^ab^	475 ± 100 ^abc^	17 ± 24 ^c^	493 ± 178 ^abc^	490 ± 23 ^abc^	ND	487 ± 111 ^abc^
e2	2-Hexenal (E)-	130 ± 38 ^abcd^	16 ± 7 ^cd^	109 ± 47 ^abcd^	129 ± 17 ^abcd^	55± 66 ^bcd^	100 ± 25 ^abc^	155 ± 35 ^abcd^	15 ± 8 ^d^	106 ± 60 ^abcd^	156 ± 9 ^abcd^	11 ± 6 ^d^	95 ± 23 ^abcd^
a4	Octanal	462 ± 167 ^abc^	57 ± 15 ^c^	736 ± 137 ^abc^	405 ± 171 ^abc^	348 ± 204 ^bc^	793 ± 8 ^abc^	593 ± 182 ^abc^	20 ± 28 ^c^	605 ± 406 ^abc^	648 ± 83 ^abc^	31 ± 6 ^c^	608 ± 229 ^abc^
e3	2-Heptenal (E)-	932 ± 21 ^abcdef^	119 ± 34 ^fg^	818 ± 80 ^abcdefg^	1114 ± 256 ^abc^	348 ± 414 ^cdefg^	828 ± 37 ^abcdefg^	1110 ± 88 ^abc^	36 ± 33 ^g^	794 ± 110 ^abcdefg^	1145 ± 311 ^abc^	56 ± 18 ^g^	953 ± 96 ^abcde^
a5	Nonanal	542 ± 233 ^abc^	81 ± 17 ^c^	711 ± 136 ^abc^	499 ± 205 ^abc^	242 ± 243 ^abc^	424 ± 599 ^abc^	731 ± 180 ^abc^	150 ± 126 ^bc^	640 ± 476 ^abc^	722 ± 89 ^abc^	40 ± 1 ^c^	588 ± 67 ^abc^
e4	2-Octenal, (E)-	1018 ± 163 ^a^	64 ± 17 ^cde^	722 ± 154 ^abcde^	1011 ± 45 ^a^	183 ± 212 ^bcde^	783 ± 60 ^abcd^	1178 ± 218 ^a^	17 ± 24 ^e^	663 ± 240 ^abcde^	1313 ± 373 ^a^	33 ± 9 ^e^	799 ± 142 ^abc^
d1	2,4-Heptadienal, (E,E)-	42 ± 18 ^a^	31 ± 6 ^a^	67 ± 18 ^a^	57± 31 ^a^	48 ± 42 ^a^	58 ± 10 ^a^	53 ± 28 ^a^	13 ± 18 ^a^	72 ± 21 ^a^	55 ± 32 ^a^	17 ± 1 ^a^	84 ± 23 ^a^
d2	2,4-Heptadienal, (E,E)-	153 ± 99 ^a^	43 ± 4 ^a^	171 ± 25 ^a^	221 ± 163 ^a^	78 ± 78 ^a^	152 ± 7 ^a^	183 ± 94 ^a^	18 ± 25 ^a^	168 ± 2 ^a^	197 ± 140 ^a^	21 ± 1 ^a^	233 ± 13 ^a^
e5	2-Nonenal, (E)-	305 ± 213 ^a^	ND	91 ± 31 ^a^	263 ± 151 ^a^	66 ± 93 ^a^	175± 148 ^a^	344 ± 221 ^a^	ND	204 ± 246 ^a^	314 ± 86 ^a^	ND	66 ± 31 ^a^
e6	2-Decenal, (E)-	485 ± 85 ^abcdef^	13 ± 18 ^ef^	677 ± 75 ^abcde^	469 ± 72 ^abcdef^	93 ± 132 ^cdef^	778 ± 118 ^ab^	597± 83 ^abcdef^	6 ± 8 ^ef^	567 ± 293 ^abcdef^	716 ± 203 ^abc^	ND	600 ± 54 ^abcdef^
d3	2,4-Nonadienal, (E,E)-	63 ± 6 ^abcd^	ND	52 ± 16 ^abcd^	73 ± 12 ^a^	6 ± 8 ^cd^	57 ± 17 ^abcd^	76 ± 4 ^a^	ND	47 ± 23 ^abcd^	92 ± 33 ^a^	ND	51 ± 14 ^abcd^
e7	2-Undecenal, (E)-	146 ± 30 ^abcde^	ND	215 ± 25 ^abcd^	150 ± 11 ^abcde^	19 ± 27 ^de^	240 ± 45 ^abc^	188 ± 29 ^abcde^	ND	180 ± 104 ^abcde^	225± 61 ^abcd^	ND	193 ± 8 ^abcde^
d4	2,4-Decadienal, (E,E)	99 ± 28 ^ab^	ND	82 ± 4 ^ab^	77 ± 40 ^ab^	5 ± 7 ^b^	82 ± 6 ^ab^	77 ± 35 ^ab^	ND	76 ± 38 ^ab^	94 ± 44 ^ab^	ND	84 ± 58 ^ab^
		Carotenoid derivatives											
c1	6-Methyl-5-hepten-2-one	90 ± 9 ^a^	126 ± 30 ^a^	85± 4 ^a^	104 ± 25 ^a^	166 ± 132 ^a^	97 ± 16 ^a^	108 ± 13 ^a^	104 ± 7 ^a^	83 ± 26 ^a^	106 ± 6 ^a^	81 ± 4 ^a^	113 ± 4 ^a^
c2	6,10-Dimethyl-5,9-undecadien-2-one,	7 ± 3 ^b^	17 ± 2 ^ab^	4 ± 1 ^b^	5 ± 0 ^b^	16 ± 10 ^ab^	4 ± 0 ^b^	6 ± 2 ^b^	8 ± 3 ^ab^	6 ± 1 ^b^	10 ± 5 ^ab^	12 ± 6 ^ab^	7 ± 0 ^b^
		Acids											
v1	Acetic acid	1233 ± 238 ^a^	2330 ± 777 ^a^	1855 ± 97 ^a^	1088 ± 10 ^a^	1859 ± 171 ^a^	1507 ± 74 ^a^	1073 ± 30 ^a^	3241 ± 727 ^a^	1503 ± 124 ^a^	1897 ± 95 ^a^	2263 ± 127 ^a^	1472 ± 1113 ^a^
v2	Butanoic acid	9 ± 1 ^a^	79 ± 82 ^a^	44± 43 ^a^	13 ± 3 ^a^	26 ± 5 ^a^	18 ± 1 ^a^	8 ± 0 ^a^	25 ± 2 ^a^	10 ± 1 ^a^	12± 4 ^a^	32 ± 1 ^a^	11 ± 4 ^a^
v3	Pentanoic acid	36 ± 22 ^a^	11 ± 7 ^a^	20 ± 6 ^a^	30 ± 11 ^a^	13 ± 9 ^a^	23 ± 0 ^a^	38 ± 22 ^a^	10 ± 2 ^a^	19 ± 13 ^a^	35 ± 4 ^a^	7 ± 1 ^a^	19 ± 0 ^a^
v4	Hexanoic acid	549 ± 327 ^a^	77 ± 30 ^a^	196 ± 129 ^a^	455 ± 156 ^a^	109 ± 59 ^a^	378 ± 79 ^a^	581 ± 212 ^a^	88 ± 23 ^a^	260 ± 224 ^a^	551 ± 43 ^a^	76 ± 21 ^a^	258 ± 35 ^a^
v5	Heptanoic acid	79 ± 103 ^a^	41 ± 16 ^a^	73 ± 103 ^a^	94 ± 124 ^a^	23 ± 25 ^a^	2 ± 2 ^a^	109 ± 147 ^a^	24 ± 27 ^a^	2 ± 3 ^a^	180 ± 247 ^a^	43 ± 9 ^a^	115 ± 75 ^a^
		Furan											
f1	2-Pentylfuran	214 ± 62 ^abc^	20 ± 8 ^de^	158 ± 27 ^abcde^	191 ± 52 ^abcd^	49 ± 63 ^cde^	140 ± 21 ^abcde^	235 ± 45 ^ab^	2 ± 3 ^e^	137 ± 64 ^abcde^	233 ± 41 ^ab^	10 ± 3 ^e^	148 ± 8 ^abcde^
		Ketones											
k1	Acetone	928 ± 70 ^abc^	1910 ± 488 ^abc^	142 ± 28 ^c^	1034 ± 485 ^abc^	1709 ± 352 ^abc^	294 ± 127 ^bc^	653 ± 636 ^abc^	1469 ± 825 ^abc^	160 ± 54 ^bc^	320 ± 128 ^bc^	2190 ± 1280 ^ab^	61 ± 18 ^c^
k2	2-Butanone	619 ± 50 ^bcde^	2259 ± 694 ^ab^	147 ± 33 ^de^	731± 169 ^bcde^	1957 ± 84 ^abc^	280 ± 18 ^cde^	472 ± 305 ^cde^	1724 ± 1053 ^abcde^	221 ± 4 ^cde^	243 ± 50 ^cde^	899 ± 596 ^bcde^	19 ± 27 ^e^
k3	1-Penten-3-one	63 ± 22 ^a^	89 ± 12 ^a^	110 ± 27 ^a^	76 ± 8 ^a^	139 ± 139 ^a^	82 ± 8 ^a^	79 ± 6 ^a^	14 ± 20 ^a^	65 ± 92 ^a^	80 ± 21 ^a^	22 ± 1 ^a^	151 ± 10 ^a^
k4	3-Penten-2-one	48 ± 68 ^a^	116 ± 106 ^a^	262 ± 318 ^a^	47 ± 54 ^a^	32 ± 28 ^a^	41 ± 12 ^a^	108 ± 4 ^a^	102 ± 122 ^a^	171 ± 82 ^a^	ND	19 ± 8 ^a^	124 ± 133 ^a^
k5	1-Octen-3-one	58 ± 4 ^abc^	15 ± 7 ^bc^	57 ± 2 ^abc^	66 ± 1 ^abc^	50 ± 59 ^abc^	60 ± 6 ^abc^	67 ± 4 ^abc^	6 ± 3 ^c^	62 ± 1 ^abc^	62 ± 5 ^abc^	12 ± 6 ^c^	69 ± 3 ^abc^
k6	3-Octen-2-one	185 ± 95 ^abc^	8 ± 11 ^c^	169 ± 59 ^abc^	191 ± 94 ^abc^	32 ± 39 ^bc^	212 ± 64 ^abc^	219 ± 98 ^abc^	ND	162 ± 114 ^abc^	212 ± 54 ^abc^	ND	161 ± 44 ^abc^
k7	3,5-Octadien-2-one	60 ± 3 ^a^	18 ± 13 ^a^	111 ± 79 ^a^	78 ± 17 ^a^	57 ± 81 ^a^	112 ± 41 ^a^	59 ± 13 ^a^	7 ± 10 ^a^	114 ± 13 ^a^	65 ± 6 ^a^	7 ± 4 ^a^	131 ± 85 ^a^
k8	3,5-Octadien-2-one, (E,E)-	100 ± 28 ^bcdef^	7 ± 1 ^ef^	130 ± 46 ^abcde^	124 ± 21 ^abcdef^	25 ± 35 ^def^	146 ± 21 ^abcd^	114 ± 26 ^abcdef^	2 ± 2 ^ef^	144 ± 29 ^abcd^	119 ± 21 ^abcdef^	1 ± 0 ^f^	179 ± 86 ^ab^
k9	2,3-Octanedione	79 ± 4 ^abc^	18 ± 0 ^c^	64 ± 33 ^abc^	75 ± 13 ^abc^	29 ± 18 ^bc^	59 ± 19 ^abc^	94 ± 7 ^abc^	19 ± 6 ^c^	79 ± 19 ^abc^	93 ± 25 ^abc^	25 ± 8 ^c^	77 ± 40 ^abc^

^abcdefg^ means within each column, samples with the same letter are not significantly different (Tukey’s test, *p* = 0.05); values are means ± SD of two biological replicates (*n* = 6); ND: Not detected. Product of bitter cassava is B, fortified is F, and sweet is S.

**Table 4:** Approximate relative µg kg^−1^ of volatile compounds quantified in lafun produced with three cassava varieties (bitter: IBA30527; fortified: IBA011371; and sweet: TMEB117) and fermented with LAB co-culture.

		*L. lactis* and *L. mesenteroides*			*W. koreensis* (Both Strains)			*W. koreensis-1* and *L. lactis*			*W. koreensis-2* and *L. mesenteroides*		
Code	Volatile Compound	B	F	S	B	F	S	B	F	S	B	F	S
		Aldehydes											
a1	Pentanal	449 ± 3 ^abc^	138 ± 79 ^bcdef^	522 ± 20 ^a^	484 ± 76 ^a^	125 ± 108 ^cdef^	474 ± 45 ^ab^	538 ± 1 ^a^	283 ± 57 ^abcdef^	524 ± 28 ^a^	452 ± 27 ^abc^	242 ± 18 ^abcdef^	550 ± 81 ^a^
a2	Hexanal	11876 ± 1382 ^a^	871 ± 1059 ^b^	13408 ±602 ^a^	14112 ±1707 ^a^	1158 ± 1523 ^b^	12518 ± 2838 ^a^	14851 ± 942 ^a^	3927 ± 1498 ^b^	14164 ± 392 ^a^	12809 ± 2082 ^a^	3202 ± 377 ^b^	14985 ± 2024 ^a^
e1	2-Pentenal (E)-	143 ± 50 ^abcd^	7 ± 10 ^cd^	231 ± 9 ^abcd^	182 ± 8 ^abcd^	ND	195 ± 4 ^abcd^	242 ± 50 ^abc^	110 ± 21 ^bcd^	248 ± 4 ^ab^	158 ± 35 ^abcd^	106 ± 1 ^bcd^	372 ± 168 ^a^
a3	Heptanal	342 ± 178 ^abc^	85 ± 120 ^bc^	592 ± 121 ^ab^	507 ± 91 ^abc^	102 ± 136 ^bc^	543 ± 238 ^abc^	474 ± 113 ^abc^	356 ± 91 ^abc^	655 ± 126 ^a^	476 ± 144 ^abc^	317 ± 8 ^abc^	794 ± 156 ^a^
e2	2-Hexenal (E)-	120 ± 52 ^abcd^	24 ± 23 ^cd^	120 ± 62 ^abcd^	171 ± 14 ^abcd^	17 ± 23 ^cd^	137 ± 54 ^abcd^	178 ± 28 ^abcd^	50 ± 4 ^abcd^	179 ± 11 ^ab^	153 ± 40 ^abcd^	47 ± 6 ^abcd^	190 ± 20 ^a^
a4	Octanal	382 ± 194 ^abc^	184 ± 107 ^c^	757 ± 255 ^abc^	625 ± 115 ^abc^	126 ± 19 ^c^	716 ± 321 ^abc^	585 ± 233 ^abc^	420 ± 70 ^abc^	977 ± 99 ^ab^	576 ± 247 ^abc^	375 ± 52 ^c^	1090 ± 221 ^a^
e3	2-Heptenal (E)-	966 ± 673 ^abcde^	150 ± 180 ^efg^	1057 ± 42 ^abc^	1313 ± 148 ^a^	157 ± 222 ^defg^	974 ± 87 ^abcd^	1187 ± 61 ^ab^	475 ± 74 ^bcdefg^	1200 ± 102 ^ab^	1010 ± 35 ^abc^	427 ± 76 ^bcdefg^	1239 ± 110 ^ab^
a5	Nonanal	443 ± 223 ^abc^	113 ± 99 ^bc^	767 ± 285 ^abc^	773 ± 116 ^abc^	151 ± 165 ^bc^	784 ± 350 ^abc^	704 ± 292 ^abc^	319 ± 53 ^abc^	1088 ± 106 ^ab^	699 ± 337 ^abc^	401 ± 107 ^abc^	1154 ± 257 ^a^
e4	2-Octenal, (E)-	888 ± 347 ^ab^	55 ± 61 ^de^	1003 ± 127 ^a^	1265 ± 104 ^a^	57 ± 80 ^de^	799 ± 252 ^abc^	1251 ± 228 ^a^	209 ± 39 ^bcde^	1082 ± 32 ^a^	1088 ± 327 ^a^	207 ± 8 ^bcde^	1189 ± 190 ^a^
d1	2,4-Heptadienal, (E,E)-	58 ± 17 ^a^	32 ± 30 ^a^	78 ± 23 ^a^	51 ± 8 ^a^	23 ± 33 ^a^	85 ± 13 ^a^	46 ± 1 ^a^	68 ± 8 ^a^	90 ± 27 ^a^	41 ± 15 ^a^	64 ± 17 ^a^	76 ± 8 ^a^
d2	2,4-Heptadienal, (E,E)-	166 ± 139 ^a^	40 ± 41 ^a^	206 ± 28 ^a^	189 ± 65 ^a^	42 ± 59 ^a^	215 ± 13 ^a^	157 ± 23 ^a^	104 ± 18 ^a^	258 ± 84 ^a^	128 ± 30 ^a^	95 ± 9 ^a^	248 ± 21 ^a^
e5	2-Nonenal, (E)-	235 ± 52 ^a^	40 ± 56 ^a^	258 ± 202 ^a^	348 ± 179 ^a^	49 ± 69 ^a^	226 ± 278 ^a^	321 ± 216 ^a^	119 ± 28 ^a^	297 ± 169 ^a^	333 ± 288 ^a^	111 ± 3 ^a^	341 ± 255 ^a^
e6	2-Decenal, (E)-	352 ± 356 ^abcdef^	24 ± 33 ^def^	812 ± 83 ^a^	654 ± 48 ^abcdef^	27 ± 37 ^def^	687 ± 362 ^abcd^	604 ± 256 ^abcdef^	136 ± 40 ^bcdef^	981 ± 120 ^a^	566 ± 255 ^abcdef^	133 ± 5 ^bcdef^	978 ± 228 ^a^
d3	2,4-Nonadienal, (E,E)-	60 ± 42 ^abcd^	ND	61 ± 5 ^abcd^	90 ± 8 ^a^	2 ± 3 ^d^	50 ± 28 ^abcd^	79 ± 15 ^a^	9 ± 5 ^bcd^	72 ± 5 ^ab^	68 ± 17 ^abc^	8 ± 4 ^cd^	77 ± 10 ^a^
e7	2-Undecenal, (E)-	109 ± 107 ^bcde^	ND	238 ± 33 ^abc^	198 ± 19 ^abcde^	ND	199 ± 112 ^abcde^	187 ± 84 ^abcde^	32 ± 11 ^cde^	288 ± 48 ^ab^	176 ± 92 ^abcde^	30 ± 1 ^cde^	325 ± 56 ^a^
d4	2,4-Decadienal, (E,E)	42 ± 46 ^ab^	ND	91 ± 0 ^ab^	111 ± 67 ^ab^	ND	101 ± 41 ^ab^	105 ± 78 ^ab^	5 ± 6 ^b^	155 ± 40 ^a^	94 ± 53 ^ab^	6 ± 1 ^b^	136 ± 23 ^ab^
		Carotenoid derivatives											
c1	6-Methyl-5-hepten-2-one	99 ± 8 ^a^	149 ± 103 ^a^	121 ± 13 ^a^	118 ± 21 ^a^	160 ± 138 ^a^	95 ± 19 ^a^	129 ± 30 ^a^	243 ± 8 ^a^	171 ± 29 ^a^	101 ± 18 ^a^	226 ± 64 ^a^	139 ± 20 ^a^
c2	6,10-Dimethyl-5,9-undecadien-2-one	9 ± 6 ^ab^	14 ± 5 ^ab^	7 ± 2 ^b^	7 ± 1 ^b^	13 ± 11 ^ab^	5 ± 2 ^b^	8 ± 1 ^ab^	25 ± 3 ^a^	11 ± 8 ^ab^	7 ± 0 ^b^	25 ± 1 ^a^	7 ± 1 ^b^
		Acids											
v1	Acetic acid	1596 ± 379 ^a^	3217 ± 1416 ^a^	1344 ± 631 ^a^	1587 ± 173 ^a^	3496 ± 2405 ^a^	1806 ± 120 ^a^	1777 ± 66 ^a^	2242 ± 361 ^a^	2070 ± 231 ^a^	1732 ± 631 ^a^	2281 ± 69 ^a^	1814 ± 193 ^a^
v2	Butanoic acid	11 ± 1 ^a^	29 ± 4 ^a^	11 ± 3 ^a^	11 ± 1 ^a^	20 ± 8 ^a^	11 ± 0 ^a^	13 ± 4 ^a^	22 ± 8 ^a^	18 ± 0 ^a^	10 ± 3 ^a^	22 ± 1 ^a^	11 ± 0 ^a^
v3	Pentanoic acid	42 ± 27 ^a^	11 ± 4 ^a^	26 ± 8 ^a^	38 ± 8 ^a^	10 ± 8 ^a^	19 ± 11 ^a^	41 ± 16 ^a^	12 ± 1 ^a^	29 ± 8 ^a^	37 ± 22 ^a^	12 ± 1 ^a^	33 ± 8 ^a^
v4	Hexanoic acid	532 ± 183 ^a^	110 ± 21 ^a^	371 ± 88 ^a^	572 ± 106 ^a^	68 ± 42 ^a^	280 ± 223 ^a^	656 ± 209 ^a^	97 ± 20 ^a^	406 ± 155 ^a^	559 ± 336 ^a^	94 ± 23 ^a^	459 ± 129 ^a^
v5	Heptanoic acid	106 ± 142 ^a^	29 ± 35 ^a^	82 ± 109 ^a^	146 ± 198 ^a^	10 ± 8 ^a^	38 ± 48 ^a^	146 ± 197 ^a^	25 ± 31 ^a^	4 ± 1 ^a^	54 ± 66 ^a^	3 ± 1 ^a^	5 ± 2 ^a^
		Furan											
f1	2-Pentylfuran	153 ± 103 ^abcde^	1 ± 1 ^e^	172 ± 12 ^abcde^	242 ± 14 ^a^	24 ± 32 ^de^	159± 58 ^abcde^	236 ± 32 ^ab^	67 ± 23 ^bcde^	205 ± 6 ^abc^	227 ± 74 ^ab^	67 ± 4 ^bcde^	241 ± 26 ^a^
		Ketones											
k1	Acetone	776 ± 954 ^abc^	1438 ± 347 ^abc^	63 ± 18 ^c^	783 ± 305 ^abc^	479 ± 445 ^abc^	226 ± 89 ^bc^	1005 ± 405 ^abc^	2508 ± 692 ^a^	264 ± 106 ^bc^	551 ± 656 ^abc^	2073 ± 495 ^abc^	88 ± 42 ^c^
k2	2-Butanone	349 ± 189 ^cde^	1448 ± 1114 ^abcde^	178 ± 86 ^de^	538 ± 32 ^bcde^	1860 ± 899 ^abcd^	385 ± 179 ^cde^	767 ± 218 ^bcde^	2747 ± 141 ^a^	366 ± 152 ^cde^	297 ± 156 ^cde^	1167 ± 195 ^abcde^	166 ± 84 ^de^
k3	1-Penten-3-one	83 ± 30 ^a^	94 ± 122 ^a^	134 ± 3 ^a^	78 ± 3 ^a^	101 ± 117 ^a^	141 ± 45 ^a^	98 ± 36 ^a^	140 ± 4 ^a^	133 ± 55 ^a^	87 ± 38 ^a^	194 ± 53 ^a^	130 ± 1 ^a^
k4	3-Penten-2-one	20 ± 11 ^a^	32 ± 29 ^a^	46 ± 65 ^a^	159 ± 93 ^a^	16 ± 3 ^a^	234 ± 29 ^a^	90 ± 6 ^a^	50 ± 31 ^a^	138 ± 71 ^a^	32 ± 45 ^a^	48 ± 20 ^a^	101 ± 78 ^a^
k5	1-Octen-3-one	61 ± 23 ^abc^	24 ± 27 ^abc^	72 ± 11 ^abc^	66 ± 11 ^abc^	18 ± 21 ^bc^	76 ± 6 ^abc^	72 ± 13 ^abc^	68 ± 11 ^abc^	90 ± 19 ^a^	69 ± 4 ^abc^	63 ± 29 ^abc^	84 ± 13 ^ab^
k6	3-Octen-2-one	199 ± 62 ^abc^	9 ± 13 ^c^	223 ± 54 ^abc^	274 ± 110 ^ab^	10 ± 14 ^c^	242 ± 41 ^abc^	268 ± 100 ^ab^	36 ± 11 ^bc^	303 ± 27 ^a^	228 ± 91 ^abc^	32 ± 4 ^bc^	285 ± 24 ^ab^
k7	3,5-Octadien-2-one	104 ± 40 ^a^	24 ± 34 ^a^	132± 48 ^a^	83 ± 1 ^a^	39 ± 54 ^a^	119 ± 10 ^a^	76 ± 12 ^a^	91 ± 16 ^a^	145 ± 33 ^a^	61 ± 13 ^a^	85 ± 22 ^a^	120 ± 16 ^a^
k8	3,5-Octadien-2-one, (E,E)-	98 ± 43 ^bcdef^	6 ± 8 ^ef^	189 ± 13 ^ab^	148 ± 13 ^abcd^	10 ± 13 ^ef^	160 ± 41 ^abc^	140 ± 28 ^abcd^	33 ± 13 ^cdef^	231 ± 62 ^a^	105 ± 7 ^abcdef^	24 ± 1 ^def^	215 ± 23 ^ab^
k9	2,3-Octanedione	67 ± 50 ^abc^	25 ± 4 ^c^	97 ± 4 ^abc^	97 ± 18 ^abc^	19 ± 6 ^c^	92 ± 31 ^abc^	87 ± 8 ^abc^	33 ± 13 ^abc^	114 ± 39 ^ab^	87 ± 1 ^abc^	26 ± 6 ^c^	117 ± 11 ^a^

^abcdefg^ means within each column, samples with the same letter are not significantly different (Tukey’s test, *p* = 0.05); values are means ± SD of two biological replicates (*n* = 6); ND: Not detected. Product of bitter cassava is B, fortified is F, and sweet is S.

**Table 5:** Approximate relative concentration of volatile compounds µg kg^−1^ (mean ± SD, *n* = 3) quantified in market samples (M1: Bodija market sample; M2: Sango market sample; M3: Mokola market sample).

		M1	M2	M3
Code	Volatile Compound			
		Aldehydes		
a1	Pentanal	256 ± 19	216 ± 10	37 ± 1
a2	Hexanal	6500 ± 396	2563 ± 130	481 ± 19
e1	2-Pentenal (E)-	38 ± 2	170 ± 127	ND
a3	Heptanal	346 ± 18	328 ± 12	65 ± 4
e2	2-Hexenal (E)-	55 ± 3	18 ± 1	ND
a4	Octanal	596 ± 40	498 ± 24	157 ± 1
e3	2-Heptenal (E)-	236 ± 14	217 ± 8	22 ± 1
a5	Nonanal	541 ± 27	810 ± 52	309 ± 28
e4	2-Octenal, (E)-	376 ± 11	119 ± 7	24 ± 0
d1	2,4-Heptadienal, (E,E)-	28 ± 4	8 ± 0	ND
d2	2,4-Heptadienal, (E,E)-	87 ± 1	ND	ND
e5	2-Nonenal, (E)-	101 ± 3	137 ± 28	29 ± 3
e6	2-Decenal, (E)-	151 ± 15	93 ± 3	0
d3	2,4-Nonadienal, (E,E)-	73 ± 2	16 ± 0	7 ± 1
e7	2-Undecenal, (E)-	ND	ND	ND
d4	2,4-Decadienal, (E,E)	8 ± 0	19 ± 1	0
		Carotenoid derivatives		
c1	6-Methyl-5-hepten-2-one	69 ± 2	252 ± 14	23 ± 1
c2	6,10-Dimethyl-5,9-undecadien-2-one,	ND	8 ± 1	4 ± 0
			Acids	
v1	Acetic acid	580 ± 83	490 ± 14	686 ± 24
v2	Butanoic acid	90 ± 3	6097 ± 99	5653 ± 82
v3	Pentanoic acid	61 ± 6	46 ± 2	210 ± 2
v4	Hexanoic acid	819 ± 16	149 ± 5	129 ± 7
v5	Heptanoic acid	421 ± 73	ND	ND
		Furan		
f1	2-Pentylfuran	223 ± 20	93 ± 9	17 ± 1
		Ketones		
k1	Acetone	29 ± 2	12 ± 0	40 ± 2
k2	2-Butanone	23 ±1	ND	ND
k3	1-Penten-3-one	22 ± 3	23 ± 2	ND
k4	3-Penten-2-one	52 ± 1	ND	ND
k5	1-Octen-3-one	21 ± 0	56 ± 0	5 ± 0
k6	3-Octen-2-one	284 ± 4	30 ± 2	11 ± 1
k7	3,5-Octadien-2-one	159 ± 23	26 ± 1	29 ± 1
k8	3,5-Octadien-2-one, (E,E)-	122 ± 22	14 ± 1	7 ± 0
k9	2,3-Octanedione	19 ± 1	14 ± 1	3 ± 0
		Alcohol		
1	1-Octen-3-ol	ND	104 ± 5	33 ± 1
		Phenols		
p1	p-cresol	ND	9 ± 1	ND
p2	m-cresol	ND	6 ± 0	ND

**Table 6:** Odour description and intensity of the volatile compounds detected by GC-O in headspace of lafun.

					Variety-Intensity ^D^	
Odour Description	Compound	LRI GC-O ^A^	LRI GC-MS ^C^	ID ^B^	M	L
green	Hexanal	800	803	A	5	5
sweet	cis-3-Hexenal	803	804	A	5	4.5
vegetable	Unknown	807		-	4	3
cheese	Butanoic acid	810		A	5.5	ND
meat	2-Methyl-3-furanthiol	865		A	4	ND
fruity	2-Heptanone	898	898	A	4.5	ND
lamb fat	cis-4-Heptenal	902	902	A	5	6.5
potato	Methional	906	908	A	ND	4
cats pee	3-Mercapto-3-methylbutanol	941		A	ND	5
fatty fruity	2-Heptenal, (E)-	955	959	A	ND	5
greenhouse	2-Methoxy-3-methylpyrazine	973	974	A	3	ND
mushroom	1-Octen-3-one	979	978	A	6	8
geranium	1,5-Octadien-3-one, (E)-	983		B	3.5	6
orange	6-Methyl-5-hepten-2-one	986	987	A	5	5
orange	Octanal	1006	1007	A	6	8
fried	2,4-Heptadienal, (E,E)-	1011	1012	A	ND	6
sharp green fuity viney	2-Hexenyl acetate, (E)-	1018		A	ND	3.5
fruity ald	Phenylacetaldehyde	1039		A	1.5	ND
greenhouses	2-Ethyl-3-methoxypyrazine	1054	1055	A	4.5	ND
fried	2-Octenal, (Z)-	1059	1059	A	5.5	7
fried	2-Octenal, (E)-	1064	1063	A	5	6
manure	4-Methylphenol (p-cresol)	1077	1077	A	5	ND
earthy coffee	2-Ethyl-3,6-dimethylpyrazine	1081	1082	A	4	4
dry earthy	2-Ethyl-3,5-dimethylpyrazine	1087	1086	A	ND	3
medicinal	Guaiacol	1091	1090	A	4	ND
fruity	3,5-Octadien-2-one, (E,Z)-	1095		B	ND	5
greenhouses, pea	2-Isopropyl-3-methoxypyrazine	1096	1096	A	4	ND
waxy, fatty	3-Nonenal, (E)-	1100		B	2.5	ND
fatty aldehyde	Nonanal	1106	1105	A	3.5	ND
fried	2,4-Octadienal, (E,Z)-	1112		A	ND	4
coriander	2-Nonenal, (Z)-	1148		A	2.5	4.5
violets	2,6-Nonadienal, (E,Z)-	1155	1154	A	6	5
waxy + medicinal	2-Nonenal, (E)-	1160	1159	A	6	6.5
parma violets	2,6-Nonadienal isomer	1169		B	6	ND
medicinal	2,4/5-Dimethylphenol	1173		B	3	ND
meat	2-Methyl-3-furyl methyl disulfide	1174	1174	A	4.5	ND
greenhouse, bell pepper	2-Isobutyl-3-methoxypyrazine	1183	1181	A	4	ND
fries	2,4-Nonadienal, (E,Z)-	1194		B	ND	5
fries	2,4-Nonadienal, (E,E)-	1214		A	6	6.5
minty	2-(2-Methylbutyl)-3-methylpyrazine	1246	1246	A	5	4
dry cardboard, earthy	Unknown pyrazine	1248			ND	4
coriander	2-Decenal, (E)	1264	1265	A	4	6.5
tea	Unknown	1273			4	2
dry cardboard, earthy	Unknown pyrazine	1279			ND	6
fatty	2,4-Decadienal, (E,Z)-	1296		B	ND	3
fried	2,4-Decadienal, (E,E)-	1318		A	8	8
fried	2,4-Decadienal isomer	1332		A	ND	3.5
coriander	2-Undecenal, (Z)-	1352		B	ND	5.5
coriander	2-Undecenal, (E)-	1367	1370	A	4.5	5.5
dry cardboard, earthy	Unknown pyrazine	1380			5.5	ND

^A^: Linear retention index on the DB-5 column, calculated from a linear equation between each pair of straight-chain alkanes C5–C25. ^B^: Linear retention index on DB-5 column, calculated from a linear equation between each pair of straight-chain alkanes C5–C25. ^C^: LRI and MS agree with those of the authentic compound; B, LRI, and MS agree with the literature value. ^D^: The average of intensities observed by two assessors for each sample (M, market sample; L, LAB sample); ND, not detected.

## Data Availability

The datasets presented in this article are not readily available because the data are part of an ongoing study. Requests to access the datasets should be directed to c.c.fagan@reading.ac.uk.
